# Effect of Ethylene Glycol: Citric Acid Molar Ratio and pH on the Morphology, Vibrational, Optical and Electronic Properties of TiO_2_ and CuO Powders Synthesized by Pechini Method

**DOI:** 10.3390/ma15155266

**Published:** 2022-07-30

**Authors:** Mónica A. Vargas-Urbano, Lorena Marín, Winny Mónica Castillo, Luis Alfredo Rodríguez, César Magén, Milton Manotas-Albor, Jesús Evelio Diosa, Katherine Gross

**Affiliations:** 1 Grupo de Transiciones de Fase y Materiales Funcionales (GTFMF), Departamento de Física, Universidad del Valle, Cali A.A. 25360, Colombia; vargas.monica@correounivalle.edu.co (M.A.V.-U.); luis.a.rodriguez@correounivalle.edu.co (L.A.R.); jesus.diosa@correounivalle.edu.co (J.E.D.); 2 Grupo CYTEMAC, Departamento de Física, Universidad del Cauca, Popayán 190003, Colombia; 3 Centro de Excelencia en Nuevos Materiales (CENM), Universidad del Valle, Cali A.A. 25360, Colombia; winny.castillo@correounivalle.edu.co (W.M.C.); katherine.gross@correounivalle.edu.co (K.G.); 4 Grupo de Película Delgadas (GPD), Universidad del Valle, Cali A.A. 25360, Colombia; 5 Instituto de Nanociencia y Materiales de Aragón (INMA), Departamento de Física de la Materia Condensada, Universidad de Zaragoza, 50009 Zaragoza, Spain; cmagend@unizar.es; 6 Laboratorio de Microscopias Avanzadas (LMA), Universidad de Zaragoza, 50018 Zaragoza, Spain; 7 Grupo de Investigación en Física Aplicada (GIFA), Departamento de Física y Geociencias, Universidad del Norte, Barranquilla 081007, Colombia; manotasm@uninorte.edu.co

**Keywords:** Pechini method, TiO_2_, CuO, band gap, optical properties

## Abstract

High-purity TiO_2_ and CuO powders were synthesized by the Pechini method, an inexpensive and easy-to-implement procedure to synthetize metal oxides. The variables of synthesis were the ethylene glycol:citric acid molar ratio and the pH. High reproducibility of the anatase and tenorite phase was obtained for all synthesis routes. The degree of purity of the powders was confirmed by XRD, FTIR, UV-Vis absorption and XPS spectra. SEM and TEM images revealed the powders are composed of micrometer grains that can have a spherical shape (only in the TiO_2_) or formed by a non-compacted nanocrystalline conglomerate. FTIR spectra only displayed vibrational modes associating TiO_2_ and CuO with nanoparticle behavior. UV-Vis absorption spectra revealed the values of maximum absorbance percentage of both systems are reached in the ultraviolet region, with percentages above 83% throughout the entire visible light spectrum for the CuO system, a relevant result for solar cell applications. Finally, XPS experiments allow the observation of the valence bands and the calculation of the energy bands of all oxides.

## 1. Introduction

Great efforts have been made over the years to control the photophysical and photochemical properties of metal oxides at the micro- and nanoscale, with the synthesis procedure being a crucial factor in controlling crystalline structure, particle size and morphology [1,2]. An ongoing quest of the scientific community is discovering the ways in which the synthesis might influence the optical and electronic properties of these materials. Metal oxide-based semiconductor materials have been obtained using different methods, including chemical vapor synthesis [3], sol-gel [4,5], hydrothermal [6], controlled precipitation [7] and polymeric precursor [8]. The polymeric precursor method, commonly known as the Pechini method, is one of the most economical and easily implemented methods to produce metal complexes from concentrated solutions of polyfunctional organic acids, salts or oxides of the cations required for the formation of metal oxides [9]; it combines low-temperature processing and versatility in the proportions of citric acid and metal cations to control the stoichiometry and the morphology of the particles and/or agglomerates, obtain compositional homogeneity and ensure low toxicity to produce a single-phase nanometric powder [10,11].

To produce multifunctional oxides such as titanium dioxide (TiO_2_) and copper II oxide (CuO) by the Pechini method, titanium tetrabutoxide and copper acetate are respectively used as concentrated solutions, and two basic chemical reactions are involved: the formation of a chelating complex composed of carboxylic acid, a chelating agent and a metal matrix, followed by its polyesterification with excess polyalcohol [10], giving rise to a viscous resin [9,11]. Continued heating of the solution causes distillation of all of the water and carboxylic acid, resulting in a polymer. The decomposition of this resin, commonly amorphous, is carried out by calcining it at temperatures below 300 °C. With this synthesis method, the problems of segregation or preferential precipitation in the solution are overcome because the cations are fixed to the resin, thus allowing greater control of the stoichiometry of the compound which is to be synthesized. The reagents commonly used in applying this method are citric acid (CA) and ethylene glycol (EG) [10,12]. Pechini reported 4:1 as the optimal EG:CA molar ratio [11]; however, these synthesis parameters have been varied to study the effect on the morphology and thus the change in the optical and electronic properties of oxides such as TiO_2_ [13], CuO [14] and ZnO [15]. The literature indicates that the structural characteristics of the semiconductor oxides can be controlled through the parameters of synthesis, depending on the technological applications desired. These include the fields of optics, electronics and telecommunications, as well as a strong representation in the field of chemistry, where they have been used as electrodes in voltaic and electrolytic cells, magnetic recording media, anode material for lithium-ion batteries, gas sensors, antibacterial agents and catalyst materials [15,16,17,18,19]. In general, the different technological applications of these materials lie in their potential to be used in photovoltaic devices [20], whose operating principle is based on the generation of electric current as a consequence of the absorption of light with an energy equal to or greater than the band gap (*E*_g_) of the material [21], requiring that the *E*_g_ value be comparable to the energy of photons of visible or ultraviolet light, that is, that it has a value of *E*_g_ < 3.5 eV [22].

In this work, we selected TiO_2_ and CuO due to their *E*_g_ values, which are found in the ultraviolet and visible spectra. There are three crystalline forms of TiO_2_: anatase, rutile and brookite. The anatase phase is metastable and has the highest photocatalytic activity, so in our study, we will synthesize and analysis this phase. It has a direct band gap transition value of 3.2 eV [23,24], although indirect band gap transitions in the range of 2.86 to 3.34 eV have also been reported [25], and the differences are attributed to variations in the stoichiometry of synthesis, impurity content, crystal size and type of electronic transition [22,25]. Tenorite (CuO) is a p-type semiconductor that has received much attention since the discovery of high-temperature cuprate superconductors. However, its electronic structure has not been fully resolved, and there are reports in the literature of direct [2,26] or indirect [26,27,28] *E*_g_ transitions. In this research, we want to evaluate whether modifications in the synthesis process (EG:CA and pH) can serve as mechanisms to finely adjust the photoelectric properties of CuO and TiO_2_ for the design of photovoltaic cells.

## 2. Materials and Methods

### 2.1. Synthesis of the CuO and TiO_2_

Powders of TiO_2_ and CuO were synthesized by the Pechini method following four different routes that involved two molar ratios of the polymerization mixture (2:1 and 4:1) and two different pHs (8 and 9), routes referred to henceforth as 2:1 pH8, 2:1 pH9, 4:1 pH8 and 4:1 pH9. In the first stage of the process, ethylene glycol (Merck, Darmstadt, Germany) was heated on a hotplate at 70 °C, and then the correct amount of citric acid was added to ensure an EG:CA molar ratio of 2:1 or 4:1, ratios that have been previously used to synthesize TiO_2_ and CuO, respectively [10,14,29]. After the citric acid completely dissolved in the ethylene glycol, the mixture was allowed to cool to room temperature, at which point the precursor of the oxide of interest was added to the mixture: Ti(OCH_2_CH_2_CH_2_CH_3_)_4_ (Titanium(IV) butoxide—Sigma-Aldrich, San Luis, MI, USA) or Cu(CO_2_CH_3_)_2_·H_2_O (Copper(II) acetate monohydrate—Merck) to produce TiO_2_ or CuO, respectively. The new mixture was maintained under constant stirring for several minutes until completely transparent to avoid phase segregation during the oxide synthesis process. To bring the mixture to a basic pH of 8 or 9, ammonium hydroxide (NH_4_OH) (Merck) was added at low stirring speed (see Figure S1, Supplementary Materials). This final mixture was heated at 140 °C on a hotplate to eliminate the solvent and favor the polyesterification reactions, which produce a resin. Finally, this resin was calcined in an oven at a temperature of 300 °C for 3 h to obtain an intermediate solid material with a high content of organic material. To determine the precalcination and sintering temperatures of the oxides, a thermal study was carried out by differential scanning calorimetry (DSC) and thermogravimetric analysis (TGA) (see Figure S2, Supplementary Materials), finding that both oxides can be crystallized with a thermal treatment at 450 °C for 4 h in an air atmosphere.

### 2.2. Characterization Techniques

Identification of the crystalline phases present in the powders of the synthesized oxides was carried out by means of the analysis of high-resolution X-ray diffractogram (XRD) scans taken in a Bruker D8 ADVANCE ECO equipment with a Cu anode (λ = 0.1540 nm); the operating voltage and current of the generator were set to 40 kV and 25 mA, respectively. The diffractogram scans were taken in powder mode, with a step size of 0.01°, in the range of 10° to 100°. To determine the morphology of the powders, scanning electron microscopy (SEM) images were taken with an FEG INSPECT-F50 instrument. A more detailed analysis, with higher spatial resolution, of the TiO_2_ and CuO grains was performed using high-resolution transmission electron microscopy (HRTEM) images, taken with an FEI-Titan Cube 60–300 microscope operated at 300 kV and equipped with a SuperTwin^®^ objective lens and a CETCOR spherical aberration corrector (CEOS company, Forserum, Sweden) that facilitates spatial resolutions of 0.08 nm.

For the qualitative determination of the functional groups, infrared spectroscopy (FTIR) experiments were performed using a Thermo Scientific Nicolet FTIR6700 infrared spectrometer to measure transmittance as a function of the wavelength of the light source, sweeping a range from 400 to 4000 cm^−1^. To determine the *E*_g_, diffuse reflectance spectra (UV-DRS) were taken at room temperature using a JASCO V-750 spectrometer equipped with an integrating sphere in a range of 200 to 2500 nm. The chemical nature and the binding energy (BE) were investigated by X-ray photoelectron spectroscopy (XPS), using an XPS Spectrometer Kratos AXIS Supra equipment.

## 3. Results

### 3.1. Structural and Morphological Analysis

#### 3.1.1. X-ray Diffraction

**Figure 1:**
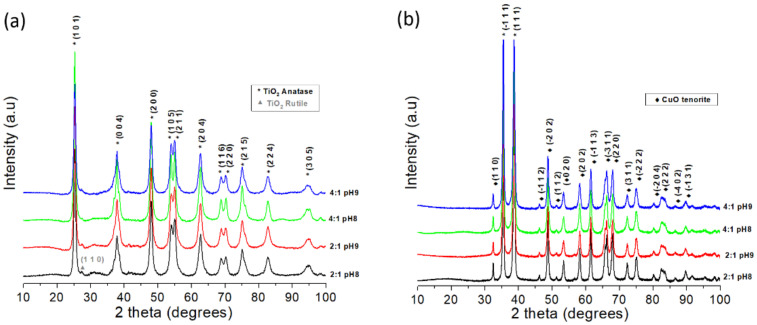
X-ray diffractograms for the (**a**) TiO_2_ and (**b**) CuO powders synthesized using the four Pechini method routes.

Figure 1 shows the powder XRDs of the synthesized TiO_2_ and CuO. All of the diffractograms present narrow and well-defined peaks, suggesting that both oxides have been synthesized with a polycrystalline character [14]. In addition, a visual comparison between the XRDs allowed us to conclude that all four synthesis routes showed the same crystalline microstructure for each type of oxide. On performing an XRD analysis by indexing the representative peaks, we found that all four synthesis routes allowed TiO_2_ to crystallize in its anatase phase (PDF 21-1272), with traces of the rutile TiO_2_ phase that can be observed in the powders synthesized with a 2:1 molar ratio. The formation of the rutile TiO_2_ phase has been previously reported using ethylene glycol:metal cation molar ratios of 3:1 and 4:1 [10]. In the case of CuO, we found that its monoclinic structure was crystallized (PDF 80-1916) and also free of secondary phases. The different routes of the Pechini method used in this work made it possible to obtain the desired phases of TiO_2_ and CuO, which appeared to be virtually pure within the resolution limits of the XRD technique.

#### 3.1.2. Scanning and Transmission Electron Microscopy

**Figure 2:**
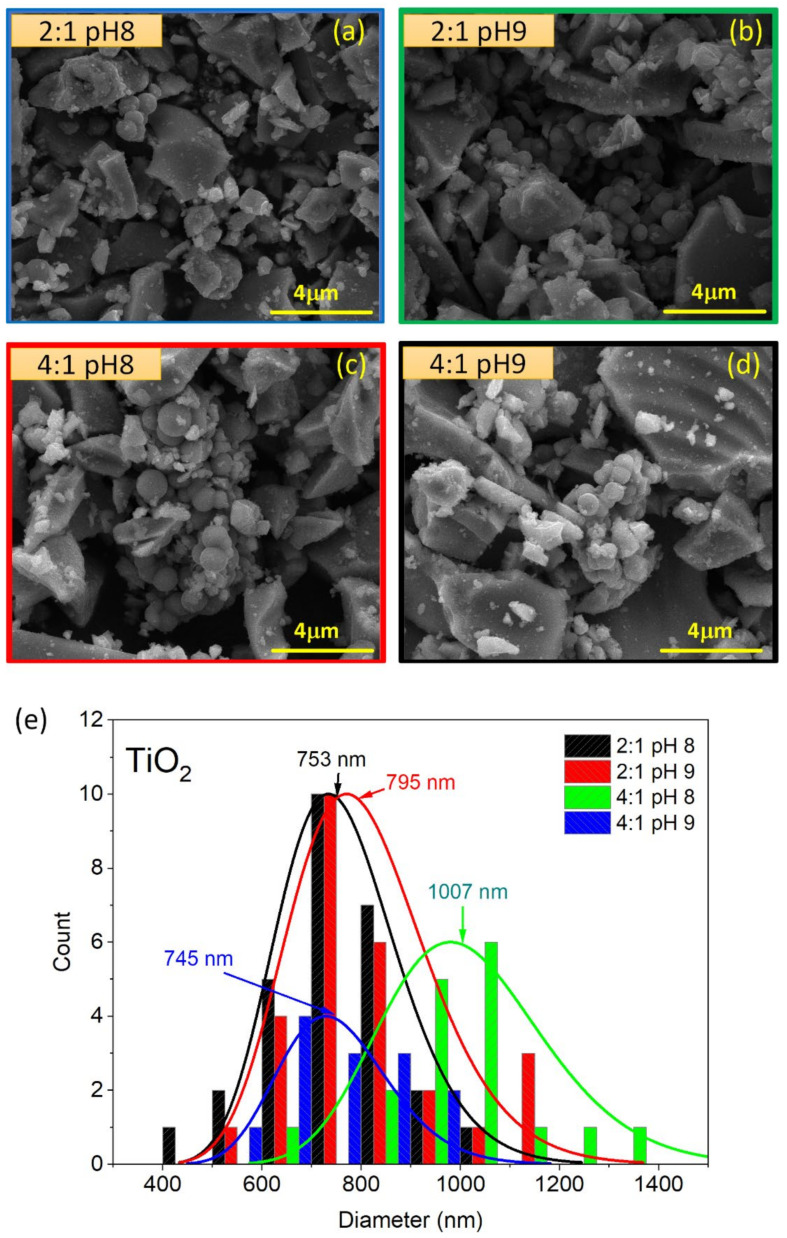
SEM images taken of TiO_2_ powders grown with the molar ratio + pH (**a**) 2:1 pH8, (**b**) 2:1 pH9, (**c**) 4:1 pH8 and (**d**) 4:1 pH9. (**e**) Histograms of diameter distributions of the spheres. Lines correspond to the lognormal distribution fittings.

SEM images taken at 20,000× magnification of synthesized TiO_2_ powders are shown in Figure 2a–d. In all cases, it is observed that TiO_2_ powders were synthesized following two main types of morphologies: (*i*) grains with irregular shapes and sizes, ranging from several microns to tens of nanometers, and (*ii*) submicron spheres. In addition to these grains, small crystallites with sizes of tens of nanometers can be observed on the grains’ surfaces. In the case of the spheres, we notice how these tend to agglomerate; some of them are perfectly defined, while others seem to be linked together. By selecting between 20 and 30 spheres from the SEM images, a diameter distribution analysis was carried out for the different routes by using a lognormal distribution function to adjust the histograms (see Figure 2c). The distribution fittings show us that the spheres grown with the 2:1 molar ratio (2:1 pH8 and 2:1 pH9) have similar mean diameters (734 and 770 nm, with standard deviations of 125 and 143 nm, respectively), which reflects that, in general, the pH variation did not produce significant changes in diameter. However, for the TiO_2_ spheres grown with the 4:1 molar ratio, we clearly see a difference between the mean diameter: while the spheres grown with pH 9 have mean sizes similar to those grown with the 2:1 ratio (745 nm, with a standard deviation of 112 nm), reducing the pH to 8 produced spheres with a larger mean diameter (1007 nm, with a standard deviation of 168 nm). Thus, the SEM analysis of the synthesized TiO_2_ reveals the resulting powders are mainly formed by micrometer- and submicrometer-sized grains, without a clear dependency on the synthesis route followed in this work. Our Pechini-based synthesis routes achieved the production of submicrospheres, similar to those synthesized by simple solution and sol-gel methods [30,31,32,33], a particular geometry that provides structural and electronic properties suitable for applications that require high absorbate binding energy for the functionalization of nanoparticles [34].

**Figure 3:**
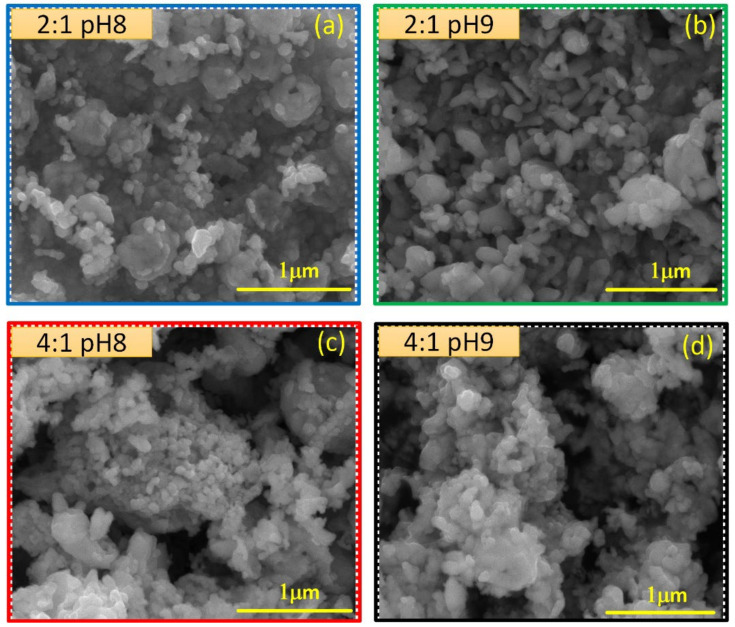
SEM images taken of CuO powders grown by following the (**a**) 2:1 pH8, (**b**) 2:1 pH9, (**c**) 4:1 pH8 and (**d**) 4:1 pH9 synthesis routes.

SEM images taken at 100,000× magnification of synthesized CuO powders are shown in Figure 3a–d. Their morphology is completely different from that observed in TiO_2_ powders. The CuO grains seem to be composed of non-compact crystallite conglomerates. A visual comparison allows us to assume that the crystallite conglomerates have larger sizes in the CuO grains grown with a 4:1 molar ratio. However, it is difficult to conclude such a fact due to their irregularity.

**Figure 4:**
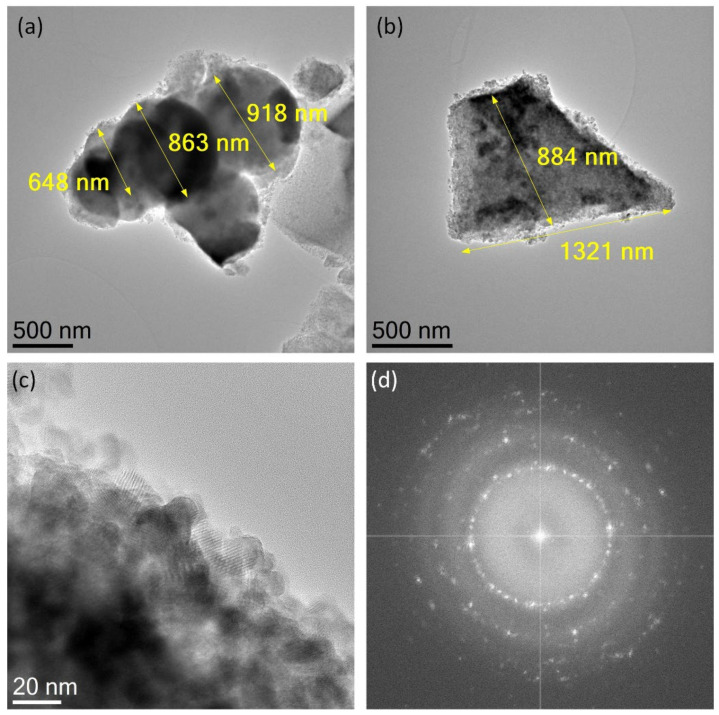
TEM images taken of (**a**) a conglomerate of submicron TiO_2_ spheres and (**b**) an irregular TiO_2_ grain crystallized by the 2:1 pH8 synthesis route. (**c**) HRTEM image recorded at the grain surface of (**b**). (**d**) Diffraction image digitally obtained from (**c**) after applying a fast Fourier transform.

**Figure 5:**
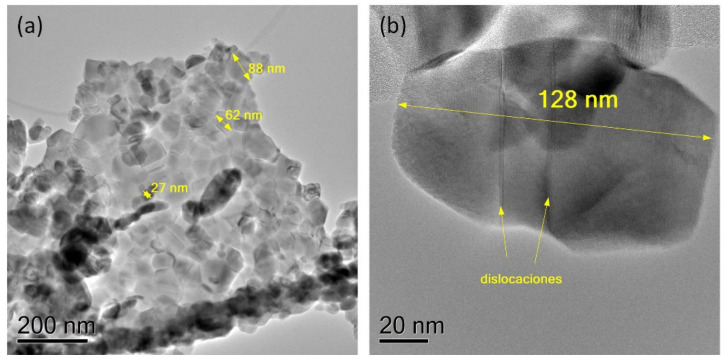
(**a**) TEM images taken of a CuO grain composed of crystallite conglomerates and synthesized by the 2:1 pH8 synthesis route. (**b**) HRTEM image taken of a CuO crystallite with dislocations.

A series of TEM images taken of representative TiO_2_ and CuO grains are displayed in Figure 4 and Figure 5. Due to the great morphological similarities of the TiO_2_ and CuO powders synthesized in each of the routes, here we will only show TEM images for TiO_2_ and CuO nanostructures obtained by the 2:1 pH8 route. Figure 4a,b show TEM images of the two types of grains observed through the SEM images of TiO_2_ powders: spherical grains with diameters between 648 and 918 nm, and an irregularly shaped grain with an average size close to 1 *μ*m. An HRTEM image, taken at the surface of the grain shown in Figure 4b, is shown in Figure 4c and reveals that the irregularly shaped grains are composed of small crystallites that are a few tens of nanometers in size. In this image, we can see that the crystallites do not exceed 20 nm in size and seem to form a non-compacted grain. A diffraction image digitally obtained from the HRTEM image in Figure 4c shows that the nanocrystallites produce diffraction rings (see Figure 4d), which allows us to conclude that they are randomly oriented along the grain. On the other hand, as we see in Figure 5a, the CuO grains are composed of crystallites that have sizes much larger than those observed for the TiO_2_ grain. Here we find, for instance, crystallites that can have average sizes greater than 100 nm. In comparison to the SEM images, we see that these nano- and submicrometer crystallites induce the formation of grains composed of conglomerates of non-compact crystallites. In some crystallites, it can be noted how a submicron size induces the formation of dislocations during the crystallization process. To illustrate this, Figure 5b shows a high-magnification TEM image of a crystallite that possesses two antiphase boundary dislocations. The crystallite size analysis carried out with TEM images is in good agreement with the results obtained by performing an estimation of the average crystallite size using the Scherrer equation on the XRDs shown in Figure 1 (see Table S1, Supplementary Materials).

### 3.2. Characterization of Vibrational Properties

#### FTIR Spectroscopy

**Figure 6:**
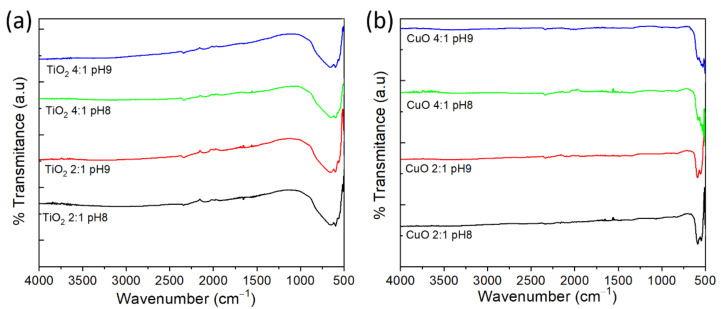
FTIR spectra for the powders of (**a**) TiO_2_ and (**b**) CuO synthesized using the four Pechini-based synthesis routes.

FTIR spectra taken from the TiO_2_ and CuO powders synthesized by the four routes are presented in Figure 6. In the case of TiO_2_, a visual inspection of the spectra indicates that there are no significant variations between them (see Figure 6a), so the vibrational properties of TiO_2_ powders are not altered by the synthesis route followed in this work. The spectra only present a broad band in the range of 500 to 1000 cm^−1^, a region in which the anatase phase of TiO_2_ shows its characteristic peaks [35,36], reflecting the high purity with which this oxide was synthesized, a result consistent with the XRD observations. Additionally, the shape of this band, wide and with two small minima associated with vibrations due to stretching of the Ti-O and Ti-O-Ti bonds, indicates that the TiO_2_ powders, despite being made up of conglomerates of nanocrystallites, have a vibrational behavior typical of nanoparticles [37,38,39,40,41,42] or nanocrystalline mesoporous powders [43], a spectrum that differs from that found in commercial powders and nanorods, which have well-defined peaks or a noisy spectrum within the range between 400 and 1000 cm^−1^ [44,45,46]. In a comparable way to TiO_2_, the FTIR spectra taken of CuO powders show only representative peaks in the range of 500 and 625 cm^−1^, a region in which CuO presents its vibrational modes, thus demonstrating its high purity and nanoparticle vibrational behavior [14]. The bands located at 547 cm^−1^ and 590 cm^−1^ related to the vibrations of the Cu–O functional group [37,47,48] are identified. While the 590 cm^−1^ band is defined for the four CuO samples, the 547 cm^−1^ band cannot be identified for the 4:1 EG:CA molar ratio (see Figure S3, Supplementary Materials).

### 3.3. Characterization of Photophysical Properties 

#### 3.3.1. UV-Vis Spectroscopy

**Figure 7:**
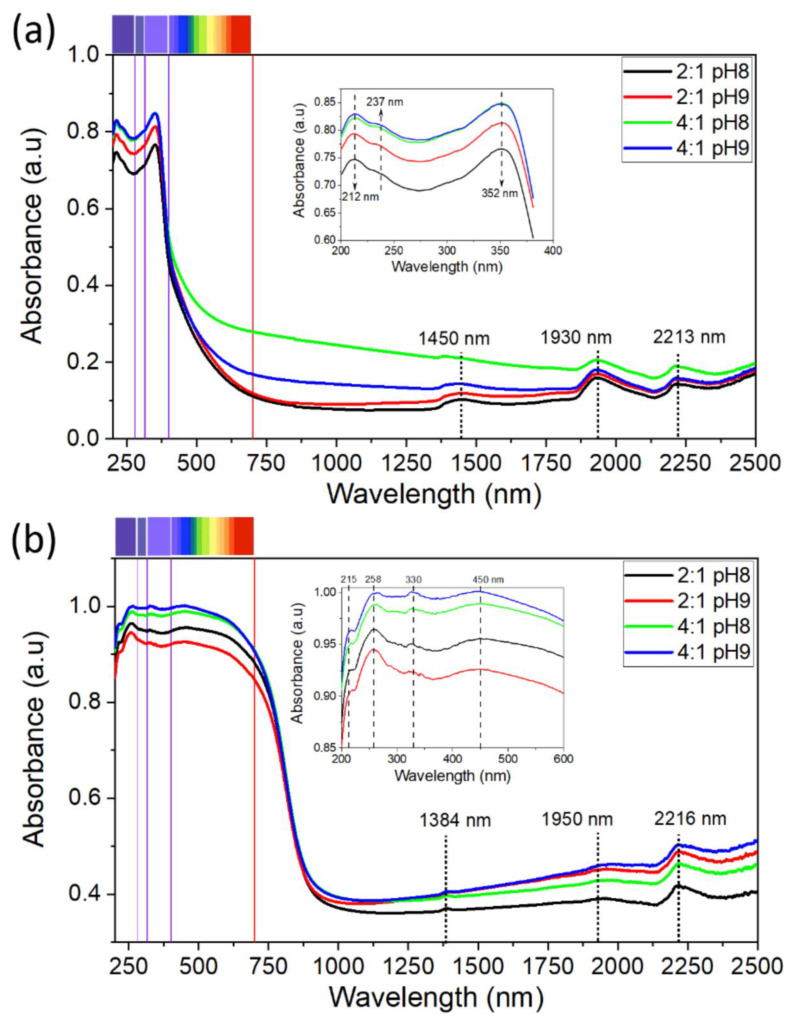
UV-Vis absorption spectra of the studied (**a**) TiO_2_ and (**b**) CuO powders. The inset of each figure is a zoom-in of the spectra in the UV region.

The optical properties of the samples were studied from ultraviolet-visible (UV-Vis) absorption spectra performed in a wavelength range from 200 to 2500 nm (See Figure 7) at room temperature. In Figure 7a, the absorption spectra for all synthesized TiO_2_ powders show that the absorbance significantly increases for values below 700 nm, reaching a maximum intensity around a wavelength of 352 nm and ranging between 75% (for 2:1 pH 8) and 84% (for 4:1 pH 9). In the UV region (inset of Figure 7a), three absorption bands around 212, 237 and 352 nm are distinguished, which are characteristic of TiO_2_ nanoparticles [49]. Moreover, we observe an increasing dependence of the absorbance percentage on the EG:CA ratio, as well as on the pH, which is remarkable for the 2:1 ratio and almost imperceptible for the 4:1 ratio. Finally, in the visible range, the TiO_2_ powders grown by the 4:1 pH8 route show the greatest absorption capacity, presenting 28% and 50% in red and violet lights, respectively, while the rest of the powders present an absorption below 15% in red light.

The UV-Vis absorption spectra of all CuO powders (see Figure 7b) exhibit the highest absorbance percentages in the visible and ultraviolet regions, reaching a maximum intensity around a wavelength of 258 nm. In the 200–450 nm region, four absorption bands around 215, 258, 330 and 450 nm are distinguished (inset of Figure 7b). In terms of the EG:CA ratio, and the pH for the 4:1 ratio, the absorption spectra of the CuO powders show a similar increasing tendency to that found in the TiO_2_, but there is decreasing tendency with the pH for the 2:1 ratio. In the visible range, all CuO samples have an absorbance greater than 83%. Finally, the absorption peaks observed around 1450–1384, 1950–1930 and 2216–2213 nm are produced by the instrument to test the spectrum curves [26,50].

**Figure 8:**
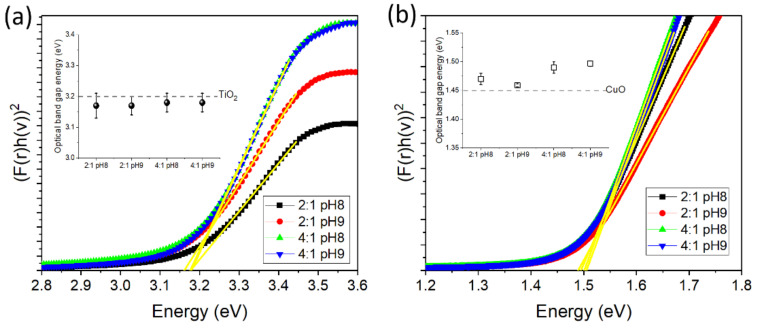
Curves of Kubelka–Munk function against absorbed light energy for the studied (**a**) TiO_2_ and (**b**) CuO powders. In the insets, a plot of *E*_g_ values as a function of the synthesis route for each oxide is shown. The dotted line in the inset of (**a**) indicates the theoretical value of bulk TiO_2_.

To extract *E*_g_ for all oxides, the Kubelka–Munk method is applied to diffuse reflectance curves obtained from the UV-Vis absorption spectra [51]. Here we plot [*f*(r)*hν*]^2^ as a function of the photon energy (*hν*), with *f*(r) being the Kubelka–Munk function (see Figure S4, Supplementary Materials). According to the linear extrapolation fits carried out on the diffuse reflectance curves, we obtained a direct *E*_g_ value for the TiO_2_ of around 3.2 eV (see Figure 8), and it was the same for all powders (see inset of Figure 8a). In the case of the direct *E*_g_ values estimated for the CuO powders, they fluctuate around 1.5 eV; while the 2:1 pH8 and 2:1 pH9 samples tend to have values slightly lower than 1.5 eV, the values for the 4:1 pH8 and 4:1 pH9 samples tend to be slightly higher. Both TiO_2_ and CuO powders showed values close to the theoretical 3.2 eV [52,53,54] and 1.45–1.57 eV [55], respectively, both showing direct band semiconductor behavior, an ideal result to improve the absorption efficiency of solar energy in photovoltaic devices [22]. Table 1 lists the optical band gap values of all samples.

#### 3.3.2. XPS Measurements

**Figure 9:**
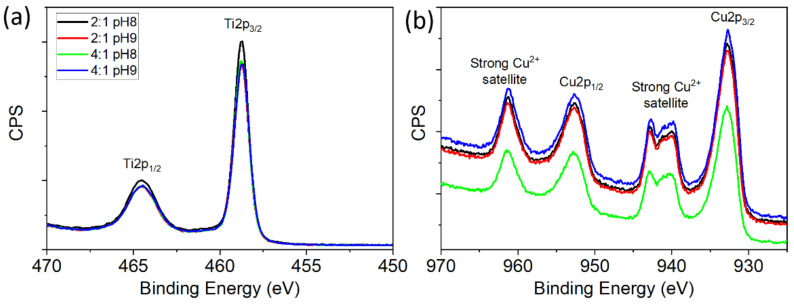
XPS spectra of the (**a**) Ti 2p and (**b**) Cu 2p peaks for the TiO_2_ and CuO powders, respectively.

To estimate the oxidation states and the valence band energy (*E*_VB_) values of the powders, XPS experiments were performed. The spectra were taken from a broad sweep of energies, from 0 to 1200 eV (see Figure S5, Supplementary Materials), but we have focused our analysis on very narrow and specific regions. Figure 9 shows high-resolution XPS spectra taken around characteristic peaks of the TiO_2_ and CuO systems. Figure 9a shows the Ti 2p peaks for the TiO_2_ system, where the expected doublet (Ti 2p_3/2_ and Ti 2p_1/2_) is detected. The BE of the peaks are 458.7 (Ti 2p_3/2_) and 464.5 eV (Ti 2p1_/2_), and they are associated with Ti(IV) oxide [56]. Figure 9b shows a comparison of the Cu 2p_1/2_ regions, observing a maximum of the Cu 2p_3/2_ signal at 933.8 eV and presenting the characteristic satellite profile reported for Cu(II). Specifically, both the BE recorded in the samples and the spectra profile correspond to what is expected for our anatase and tenorite phases [57]. In both oxides, there is no spectra variation with the synthesis routes.

**Figure 10:**
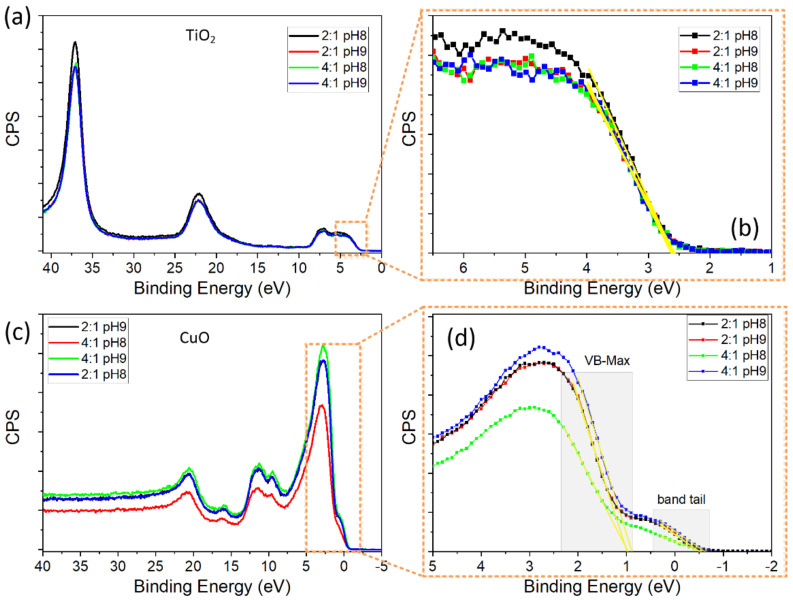
XPS spectra of (**a**) TiO_2_ and (**c**) CuO powders taken around a low-binding energy region. (**b**,**d**) are the XPS valence band spectra of the oxides extracted from (**a**,**c**), respectively.

Figure 10 shows the XPS spectra of the low-binding-energy electrons as well as XPS valence band spectra of all powders. While the TiO_2_ powders exhibit a valence band with a well-defined maximum energy edge, located above the Fermi level (*E*_F_, it has BE = 0), the CuO powders present a distorted valence band with a small band tail that has a negative energy edge. The presence of a band tail in the XPS valence band spectra of CuO has been previously reported [58,59,60,61]; it is caused by defects in disordered regions of the system, mainly located at the surface, that promote electron hybridization. From the XPS valence bands, we estimated *E*_VB_ by performing a linear extrapolation to zero counts per second (CPS) around the maximum energy edge. All synthesized TiO_2_ powders have the same *E*_VB_ of 2.6 eV, a value that is in good agreement with that reported in the literature [62]. On the other hand, most of the CuO powders have a maximum *E*_VB_ (*E*_VB-max_) of around 0.98 eV and a minimum *E*_VB_ (*E*_VB-min_) close to −0.51 eV, the latter determined from the band tail. Only the CuO powder synthesized by the 4:1 pH9 route presented lower values of *E*_VB-max_ (0.89 eV) and *E*_VB-min_ (−0.616 eV).

**Figure 11:**
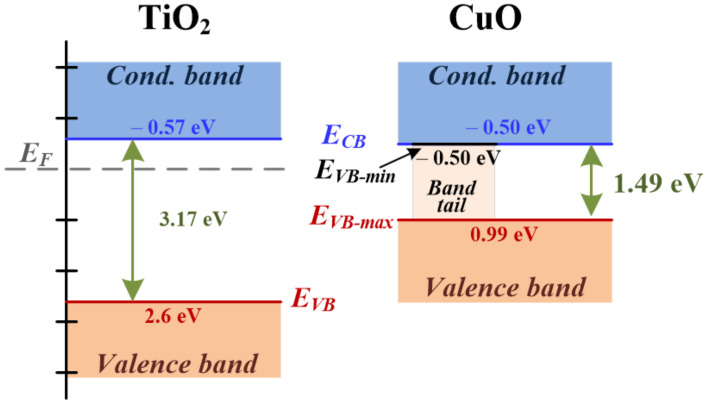
Schematic diagram of the energy bands for the TiO_2_ and CuO powders. The values of the energy levels correspond to the powders synthesized by following the 2:1 pH8 route.

From the *E*_g_ and *E*_VB_ values previously estimated, we calculate the conduction band energy (*E*_CB_) (*E*_CB_ = *E*_VB_ − *E*_g_ [63]) values that are listed in Table 1. Thus, we have the full information of the energy bands of our powders. According to Table 1, all TiO_2_ powders have a similar *E*_CB_ of around −0.58 eV, a value that is lower than that found in most of the CuO powders (~−0.51 eV). Similar to *E*_VB_, the CuO powder synthesized by the 4:1 pH9 route presents a lower *E*_CB_ (−0.62 eV). It is important to note that the values of *E*_CB_ for the CuO powders are slightly higher than the minimum *E*_VB_ and, as a consequence, show an overlapping effect between the valence and conduction bands. From the electrical point of view, this overlapping induces a metal behavior in some regions placed at the grain surface of the CuO system. This metallic behavior observed at the surface of the semiconductor oxide can be used to trap photogenerated electrons, preventing a rapid electron-hole recombination, a useful mechanism to improve the photocatalytic activity of this system [64]. In Figure 11, we present a diagram of the energy bands for the TiO_2_ and CuO powders synthesized by the 2:1 pH8 route, which represent the general behavior of the studied oxides.

**Table 1:** Values of *E*_g_, *E*_VB_ and *E*_CB_ for all powders. The values labeled by * and ** correspond to the *E*_VB-max_ and *E*_VB-min_, respectively, for the CuO.

Sample	*E*_g_ (eV)	*E*_VB_ (eV)	*E*_CB_ (eV)
2:1 pH8—TiO_2_	3.17 ± 0.04	2.6 ± 0.1	−0.57 ± 0.07
2:1 pH9—TiO_2_	3.17 ± 0.03	2.6 ± 0.1	−0.57 ± 0.08
4:1 pH8—TiO_2_	3.18 ± 0.03	2.6 ± 0.2	−0.6 ± 0.1
4:1 pH9—TiO_2_	3.18 ± 0.03	2.6 ± 0.1	−0.58 ± 0.07
2:1 pH8—CuO	1.494 ± 0.003	0.99 ± 0.06 * −0.504 ± 0.005 **	−0.50 ± 0.03
2:1 pH9—CuO	1.489 ± 0.004	0.99 ± 0.05 * −0.51 ± 0.01 **	−0.50 ± 0.03
4:1 pH8—CuO	1.502 ± 0.004	0.97 ± 0.03 * −0.55 ± 0.01 **	−0.53 ± 0.02
4:1 pH9—CuO	1.51 ± 0.01	0.89 ± 0.07 * −0.616 ± 0.005 **	−0.62 ± 0.04

## 4. Conclusions

High-purity TiO_2_ and CuO powders were synthesized following four Pechini-method-based synthesis routes. From the crystalline structure point of view, the variables of synthesis used in this work—the EG:CA ratio (2:1 and 4:1) and pH (8 and 9)—enabled the production of single-phase powders of the anatase TiO_2_ and tenorite CuO systems, without the detection of secondary phases, demonstrating that all routes enable the synthesis of high-purity oxides in the desired phases. The microscopic analysis showed the synthesized powders are formed of grains with micrometer and submicrometer sizes; those grains with irregular shapes are composed of non-compact crystallite conglomerates. In the case of TiO_2_ powders, well-defined submicron spheres were synthesized with similar diameters in three of the synthesis routes (of around 750 nm for 2:1 pH8, 2:1 pH9 and 4:1 pH9). The high purity of the synthesized powders was also supported by FTIR experiments, in which the spectra only defined the characteristic peaks of the anatase TiO_2_ and tenorite CuO phases. In addition, the formation of a wide band around the Ti-O, Ti-O-Ti and Cu-O vibration signals indicates the powders have a nanoparticle vibratory behavior, so we could infer that the non-compacted arrangement of the nanocrystallites that form the irregular grains governs the vibrational properties of the powders.

Although the crystalline microstructure and morphological and vibrational properties of the powders did not show a clear dependency on the synthesis routes for each oxide, some differences were observed in the electronic and optical properties. While all synthesized TiO_2_ powders had an *E*_g_ of approximately 3.2 eV, the optical band gap values of the CuO powders presented variations that depend on the synthesis route: the 2:1-ratio-based route promotes a slightly lower *E*_g_ (~1.49 eV) compared to the 4:1-ratio-based one (~1.51 eV), with the 2:1 pH9 route being the one that produces the lowest *E*_g_ (1489 eV). Both TiO_2_ and CuO powders have a direct band gap and absorb in the ultraviolet and visible regions, which is required for applications that use solar energy as an excitation source for the electronic transitions of semiconductor oxides. TiO_2_ shows its maximum absorption in the ultraviolet region and decreases exponentially when entering the visible region. The 4:1 pH8 ratio shows the best response in the visible region, with values of 50% and 28% for the violet and red lights, respectively. The CuO powders show promising results due to their absorbance percentages being high along the ultraviolet and visible region, above 85%. Finally, analysis of the XPS valence band spectra reveals that the CuO powders can exhibit a hybrid metal–semiconductor behavior due to the presence of a band tail in the valence band that overlaps the conduction band. This is not the case for the TiO_2_ powders, where a well-defined valence band indicates a semiconductor behavior. Therefore, the synthesis routes used in this work allows for the preparation of high-purity TiO_2_ and CuO powders with similar morphological characteristics, but they finely adjust the photophysical properties relevant to the design of photovoltaic cells.

## Data Availability

Not applicable.

## Supplementary Materials

The following supporting information can be downloaded at: https://www.mdpi.com/article/10.3390/ma15155266/s1, Figure S1: pH as a function of NH_4_OH (a) TiO_2_ and (b) CuO; Figure S2: Results of the DTA and TGA thermal analysis of (a) 2:1 pH8 TiO_2_ and (b) 4:1 pH8 CuO; Figure S3: IR spectra zoom for the sample CuO; Figure S4: Estimation of the direct band gap (a) TiO_2_ and (b) CuO; Figure S5: Wide energy range X-ray photoelectron spectroscopy (XPS) spectra for (a) TiO_2_ and (b) CuO; Table S1: Average crystallite size values using the Scherrer formule.
