# Supplementary material for: Effect of Ethylene Glycol: Citric Acid Molar Ratio and pH on the Morphology, Vibrational, Optical and Electronic Properties of TiO2 and CuO Powders Synthesized by Pechini Method
Source: Materials (Basel). 2022 Jul 30;15(15):5266. doi: 10.3390/ma15155266 (PMC9369947; doi:10.3390/ma15155266)
Supplement: Supplementary file 1 [file materials-15-05266-s001.zip › materials-1743494-supplementary.pdf]

Effect of Ethylene Glycol:Citric Acid Molar Ratio and pH on the Morphology, Vibrational, Optical and Electronic Properties of TiO_2 and CuO Powders Synthesized by Pechini Method

Mónica A. Vargas-Urbano [1,2], Lorena Marín [3,4,*], Winny Mónica Castillo [3], Luis Alfredo Rodríguez [1,3], César Magén [5,6], Milton Manotas-Albor [7], Jesús Evelio Diosa [1,3] and Katherine Gross [3,4]

[1] Grupo de Transiciones de Fase y Materiales Funcionales (GTFMF), Departamento de Física, Universidad del Valle, A.A. 25360 Cali, Colombia; vargas.monica@correounivalle.edu.co (M.A.V-U.); luis.a.rodriguez@correounivalle.edu.co (L.A.R.); jesus.diosa@correounivalle.edu.co (J.E.D.)
[2] Grupo CYTEMAC, Departamento de Física, Universidad del Cauca, Popayán, Colombia; monicavargas@unicauca.edu.co (M.A.V-U.)
[3] Centro de Excelencia en Nuevos Materiales (CENM), Universidad del Valle, A.A. 25360 Cali, Colombia; marin.lorena@correounivalle.edu.co (L.M.); winny.castillo@correounivalle.edu.co (W.M.C.); luis.a.rodriguez@correounivalle.edu.co (L.A.R.); katherine.gross@correounivalle.edu.co (K.G.); jesus.diosa@correounivalle.edu.co (J.E.D.)
[4] Grupo de Película Delgadas (GPD), Universidad del Valle, A.A. 25360 Cali, Colombia; marin.lorena@correounivalle.edu.co (L.M.); katherine.gross@correounivalle.edu.co (K.G.)
[5] Instituto de Nanociencia y Materiales de Aragón (INMA), Departamento de Física de la Materia Condensada, Universidad de Zaragoza, 50009 Zaragoza, Spain; cmagend@unizar.es (C.M.)
[6] Laboratorio de Microscopias Avanzadas (LMA), Universidad de Zaragoza, 50018 Zaragoza, Spain; cmagend@unizar.es (C.M.)
[7] Grupo de Investigación en Física Aplicada (GIFA), Departamento de Física y Geociencias, Universidad del Norte, Barranquilla, Colombia; manotasm@uninorte.edu.co (M. M-A)
* Correspondence: marin.lorena@correounivalle.edu.co; Tel.: +57 3167696970

Supplementary materials:

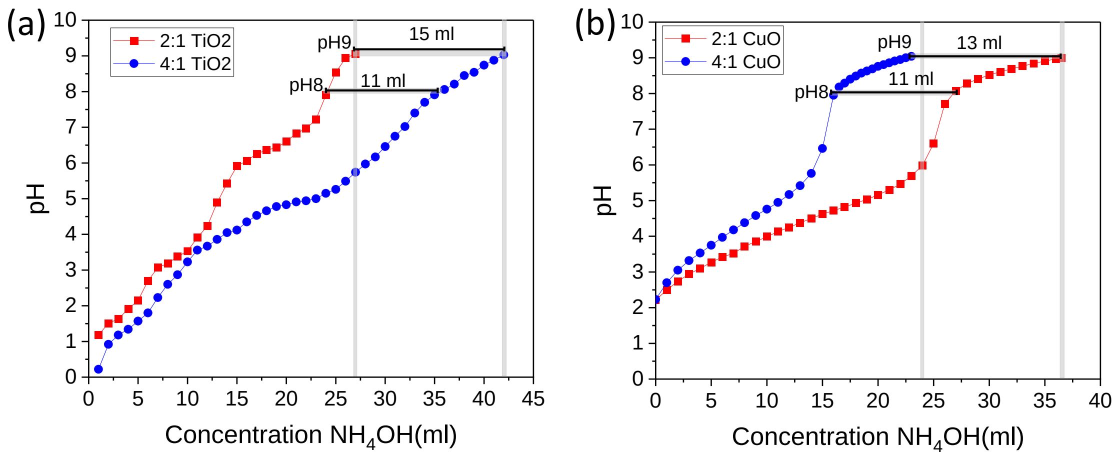

Figure S1. pH as a function of NH_4OH (a) TiO_2 and (b) CuO

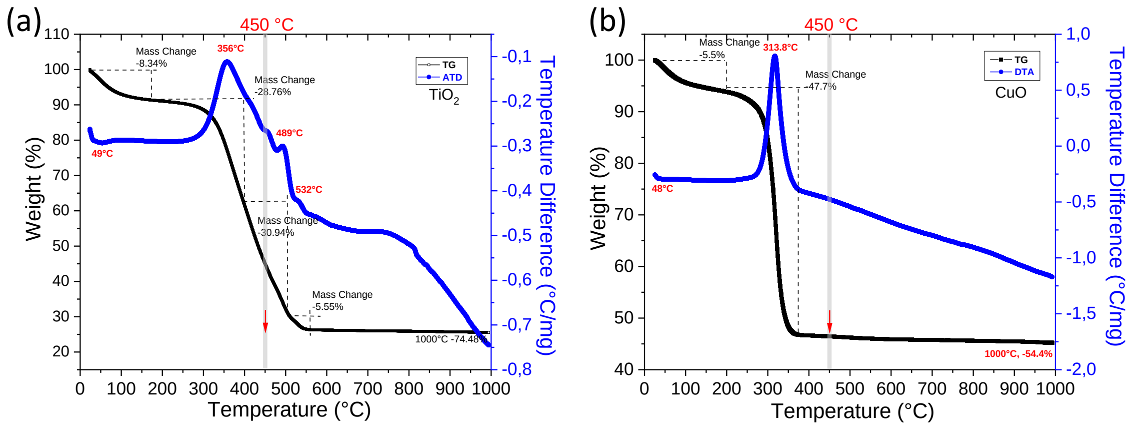

Figure S2. Results of the DTA and TGA thermal analysis of (a) 2:1 pH8 TiO_2 and (b) 4:1 pH8 CuO

Table S1. Average crystallite size values using the Scherrer formule.

Sample	Crystallite size (nm)
2:1 pH8 – TiO_2	11 ± 4
2:1 pH9 – TiO_2	12 ± 4
4:1 pH8 – TiO_2	10 ± 4
4:1 pH9 – TiO_2	10 ± 4
2:1 pH8 – CuO	29 ± 9
2:1 pH9 – CuO	24 ± 6
4:1 pH8 – CuO	21 ± 5
4:1 pH9 – CuO	21 ± 5

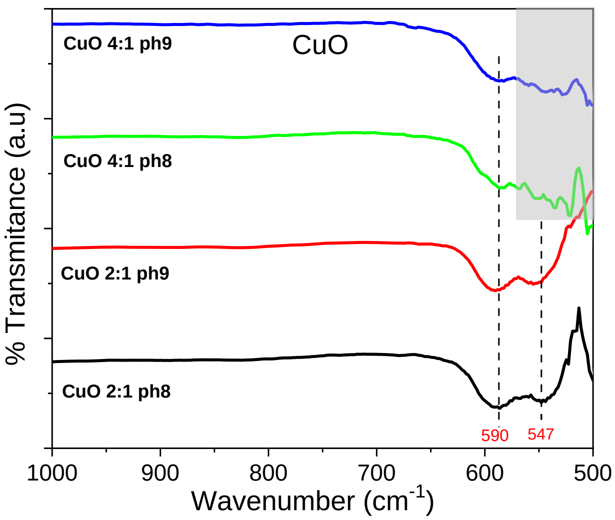

Figure S3. IR spectra zoom for the samples of CuO

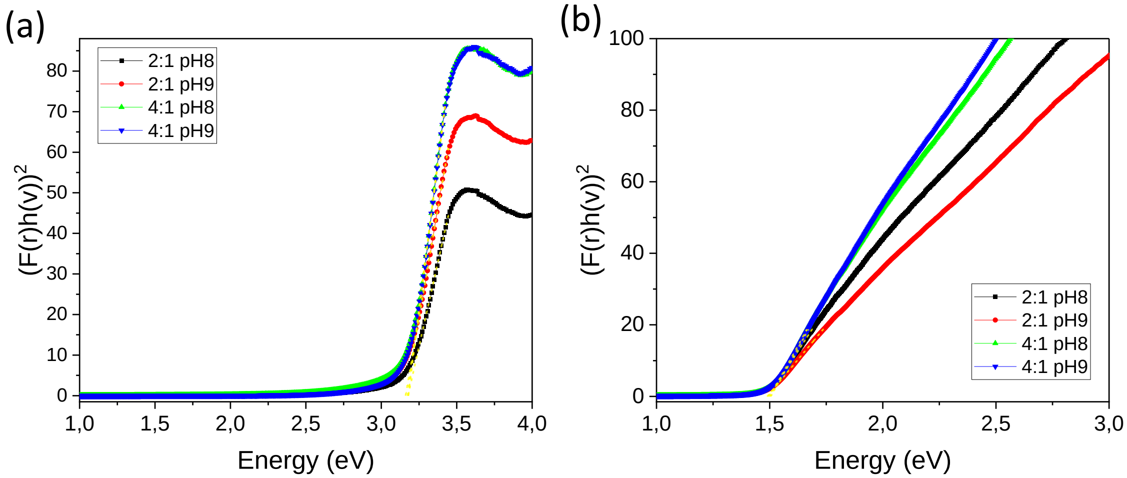

Figure S4. Estimation of the direct band gap (a) TiO_2 and (b) CuO

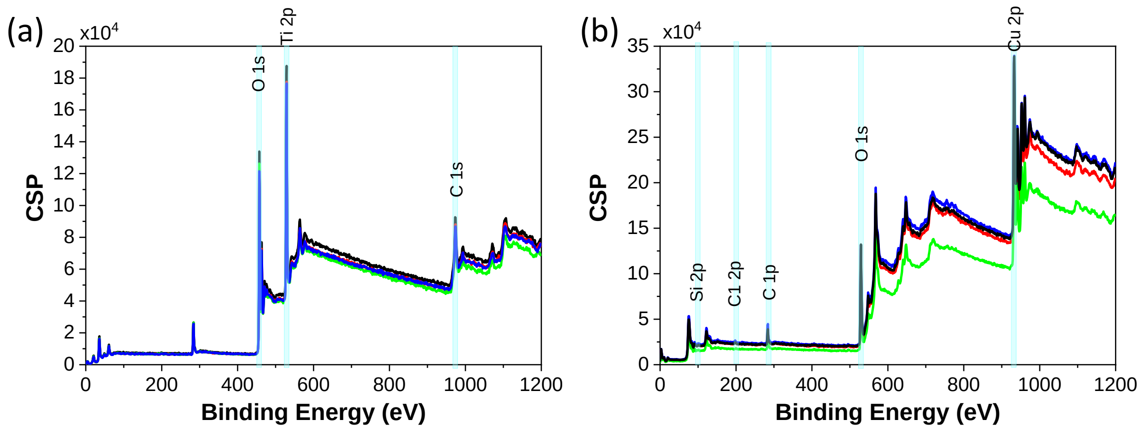

Figure S5. Wide energy range X-ray photoelectron spectroscopy (XPS) spectra of (a) TiO_2 and (b) CuO.
